# Temporal dynamics and metagenomics of phosphorothioate epigenomes in the human gut microbiome

**DOI:** 10.1186/s40168-025-02071-4

**Published:** 2025-03-24

**Authors:** Shane R. Byrne, Michael S. DeMott, Yifeng Yuan, Farzan Ghanegolmohammadi, Stefanie Kaiser, James G. Fox, Eric J. Alm, Peter C. Dedon

**Affiliations:** 1 Department of Biological Engineering, Massachusetts Institute of Technology, Cambridge, MA USA; 2 Center for Environmental Health Sciences, Massachusetts Institute of Technology, Cambridge, MA USA; 3 Pharmaceutical Chemistry, Goethe University, Frankfurt, Germany; 4 Division of Comparative Medicine, Massachusetts Institute of Technology, Cambridge, MA USA; 5 Center for Microbiome Informatics and Therapeutics, Massachusetts Institute of Technology, Cambridge, MA USA; 6 Infectious Disease and Microbiome Program, Broad Institute of MIT and Harvard, Cambridge, MA USA; 7 Singapore-MIT Alliance for Research and Technology, Antimicrobial Resistance IRG, Singapore, Singapore

**Keywords:** Human gut microbiome, Mouse gut microbiome, Phosphorothioate, Epigenetics, Mass spectrometry, PT-seq, Metagenomic analysis

## Abstract

**Background:**

Epigenetic regulation of gene expression and host defense is well established in microbial communities, with dozens of DNA modifications comprising the epigenomes of prokaryotes and bacteriophage. Phosphorothioation (PT) of DNA, in which a chemically reactive sulfur atom replaces a non-bridging oxygen in the sugar-phosphate backbone, is catalyzed by *dnd* and *ssp* gene families widespread in bacteria and archaea. However, little is known about the role of PTs or other microbial epigenetic modifications in the human microbiome. Here we optimized and applied fecal DNA extraction, mass spectrometric, and metagenomics technologies to characterize the landscape and temporal dynamics of gut microbes possessing PT modifications.

**Results:**

Exploiting the nuclease-resistance of PTs, mass spectrometric analysis of limit digests of PT-containing DNA reveals PT dinucleotides as part of genomic consensus sequences, with 16 possible dinucleotide combinations. Analysis of mouse fecal DNA revealed a highly uniform spectrum of 11 PT dinucleotides in all littermates, with PTs estimated to occur in 5–10% of gut microbes. Though at similar levels, PT dinucleotides in fecal DNA from 11 healthy humans possessed signature combinations and levels of individual PTs. Comparison with a widely distributed microbial epigenetic mark, m^6^dA, suggested temporal dynamics consistent with expectations for gut microbial communities based on Taylor’s Power Law. Application of PT-seq for site-specific metagenomic analysis of PT-containing bacteria in one fecal donor revealed the larger consensus sequences for the PT dinucleotides in Bacteroidota, Bacillota (formerly Firmicutes), Actinomycetota (formerly Actinobacteria), and Pseudomonadota (formerly Proteobacteria), which differed from unbiased metagenomics and suggested that the abundance of PT-containing bacteria did not simply mirror the spectrum of gut bacteria. PT-seq further revealed low abundance PT sites not detected as dinucleotides by mass spectrometry, attesting to the complementarity of the technologies.

Video Abstract

**Conclusions:**

The results of our studies provide a benchmark for understanding the behavior of an abundant and chemically reactive epigenetic mark in the human gut microbiome, with implications for inflammatory conditions of the gut.

**Supplementary Information:**

The online version contains supplementary material available at 10.1186/s40168-025-02071-4.

## Introduction

There are now dozens of enzymatically installed DNA modifications—the epigenome—in all forms of life, with the greatest diversity occurring in prokaryotes and bacteriophage [1]. While bacterial DNA modifications are best known for restriction-modification (RM) systems, they are now also known to regulate gene expression [2–10]. Phosphorothioation (PT) of DNA, in which a sulfur atom replaces a non-bridging oxygen in the sugar-phosphate backbone, is the only known naturally occurring DNA backbone modification. Developed more than 50 years ago as a synthetic modification to engineer nuclease resistance into oligonucleotides [11], we more recently discovered that PTs occur naturally in the genomes of bacteria [12] and archaea [13]. To date, PTs have only been detected in prokaryotes and only in DNA [14].

Synthesis of PTs is so far known to be mediated by three gene families—dndABCDE, sspBCD, and brxPCZL (BREX type 4)—whether functioning in RM or regulating gene expression [12, 13, 15–18]. As atypical RM systems, DndFGHI and SspFGH function as restriction endonuclease complexes to cleave unmodified “non-self” DNA [13, 16, 19, 20] in spite of only ~ 10–15% of available consensus sequences being modified with PT [21], while variant SspE and BREX type 4 systems possess antiviral activity without restriction endonuclease activity [15, 17]. Similar to methylation-based restriction-modification systems, the Dnd and Ssp modification proteins catalyze phosphorothioation on one or both DNA strands of specific consensus sequences. For example, DndABCDE modifies both DNA strands at 5′-G*AAC-3′/5′-G*TTC-3′ sequences (where “*” denotes a PT linkage) in *Escherichia coli* B7A and 5′-G*GCC-3′/5′-G*GCC-3′ in *Pseudomonas fluorescens* pf0-1 [16, 21, 22], while PTs occur as a single-strand modification at C*CA in *Vibrio cyclitrophicus* FF75 [15, 21].

An unusual feature of PT modification systems is that many PT-containing bacteria lack restriction genes, which suggests an alternative epigenetic role of PTs [21, 23]. For example, PTs have been proposed to provide epigenetic regulation of transcription of redox homeostasis genes [16], which may relate to a signaling function for the easily oxidized and nucleophilic sulfur in PTs. Indeed, there is evidence that PTs provide some protective effects in cells exposed to reactive oxygen and nitrogen species, such as peroxides [16, 24] and peroxynitrite [25]. Contrasting with this protection, PT-containing bacteria are fivefold more sensitive to neutrophil-derived hypochlorous acid (HOCl) due to extensive DNA breaks at PT sites [26]. Given the widespread distribution of PT gene systems among bacteria and archaea and a preliminary report by Sun et al. [27] of PTs present in human fecal DNA, the reactivity of PTs with these chemical mediators of inflammation raises questions about how PT-containing microbes might behave in the healthy gut microbiome or be altered by chronic inflammation of the gut, such as inflammatory bowel disease (IBD) [28, 29].

Here we undertook a foundational analysis of PT epigenetics in the human gut microbiome by exploring the landscape and temporal dynamics of PT dinucleotides in fecal DNA from 11 healthy humans, using an enhanced fecal DNA extraction method and optimized chromatography-coupled mass spectrometry (LC–MS). Data for PTs were compared to a widespread and well-established microbial epigenetic mark, m^6^dA. We also performed a metagenomic analysis of PT-containing microbes in fecal DNA using a novel NGS technology—PT-seq. With results revealing that 5–10% of gut microbes possess PTs, our studies lay the groundwork for future investigations of the role of PTs in IBD and other diseases.

## Results

### Optimizing the analytical and informatic platform for PT analysis in human fecal DNA

**Fig. 1:**
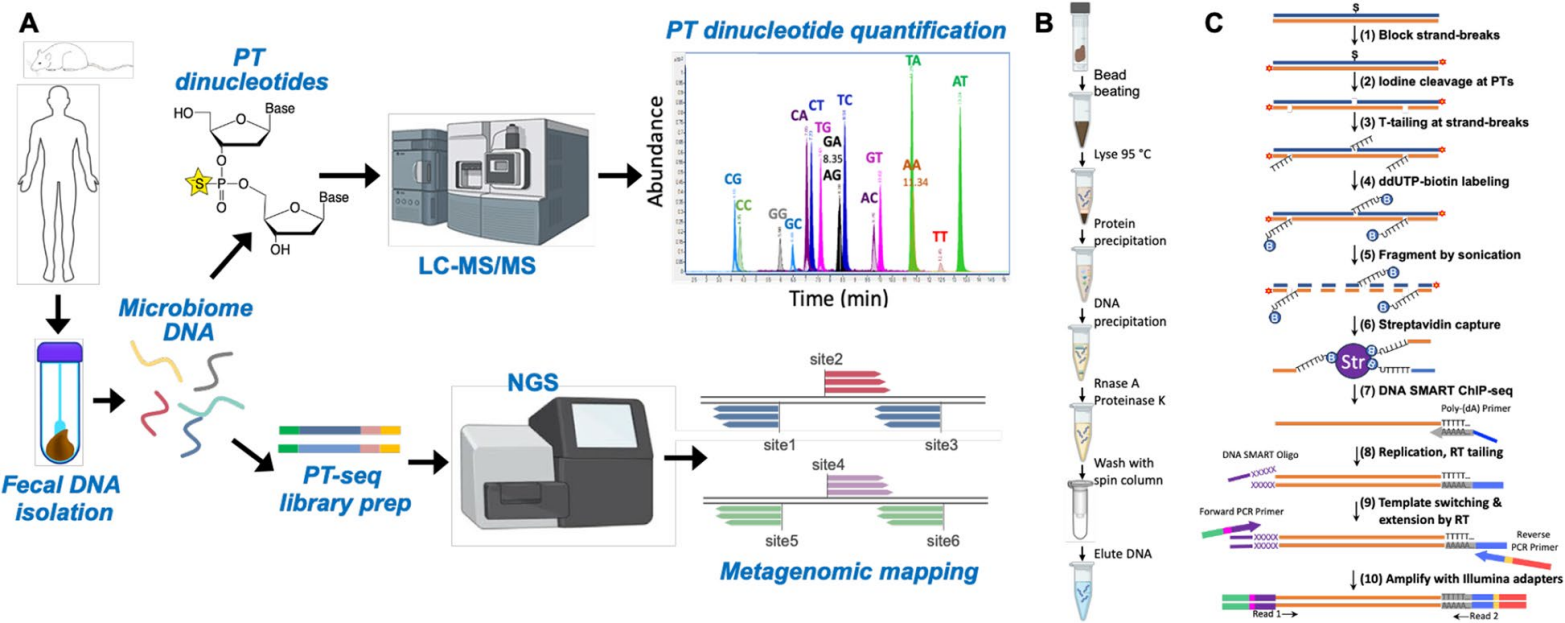
Workflows and optimized technologies for identifying and quantifying PT dinucleotides and mapping PT sites in gut microbes. **A** Purified fecal DNA was analyzed for PT dinucleotide content by LC–MS and subjected to PT-seq for metagenomic analysis of bacterial identity and PT consensus sequence. **B** Workflow for optimized fecal DNA isolation and purification, which improved yield by tenfold. **C** Workflow for optimized PT-seq. The streptavidin capture step significantly reduced the level of noise and increased the read pileup sensitivity

While PT modification gene systems are widespread among bacteria and archaea [13, 15, 16, 18, 30] and there is evidence for PTs in human gut microbes [27], there has not been a systematic study of the true breadth, depth, and behavior of PT epigenetics in the gut microbiome. We initiated this exploration by developing a workflow (Fig. 1A) for identifying and quantifying PT dinucleotide spectra and PT-containing microbes in fecal DNA. Here we optimized existing technologies to improve yields of fecal DNA extraction (Fig. 1B) [31], to increase the sensitivity and specificity of chromatography-coupled mass spectrometry (LC–MS) for quantifying PT dinucleotides [30], and to increase read depth for next generation sequencing (NGS)-based metagenomic analysis of the hundreds of genomes present in fecal DNA (Fig. 1C) [32]. The relatively low efficiency of fecal DNA extraction with the commercial Qiagen QIAmp Fast DNA Stool Mini Kit (Supplementary Fig. S1A) and the modest increase with the International Human Microbiome Standards (IHMS) Protocol Q [33, 34] (4–8 μg DNA/200 mg fecal material; Supplementary Fig. S1A), which extracted DNA from only 5% of a diluted sample, raised concerns about wasting samples and resources and the potential for biased microbiome representation caused by less efficient DNA extraction, while the harsh conditions used in Protocol Q raised concerns about PT stability during DNA isolation. To improve DNA yields, we revised the protocol by (1) avoiding the initial PBS dilution and homogenization steps and directly mixing fecal material with QIAamp InhibitEx buffer and (2) increasing the concentration of fecal material in the dilution buffer. Supplementary Fig. S1B shows that these changes increased the yield of DNA per mg of feces by more than tenfold with a linear dose–response. The necessity of the bead-beating step is demonstrated in the fivefold decrease in DNA yield in the absence of beads (Supplementary Fig. S1C). We further modified Protocol Q to increase the purity of the extracted DNA by adding an RNase A treatment to remove unwanted nucleic acid contamination and a second wash with buffer AW2 during the final cleanup steps before spin column elution. Overall, the optimized Protocol Q significantly outperformed the QIAmp Fast DNA Stool Kit in terms of DNA yield (Supplementary Fig. S1D), *A*_260_/*A*_230_ purity (Supplementary Fig. S1E), and *A*_260_/*A*_280_ purity (Supplementary Fig. S1F). Evidence for this improved purity is shown in Supplementary Fig. S1H, with the optimized Protocol Q resulting in lower levels of UV-detectable contamination in fecal DNA digests resolved by HPLC.

### A uniform spectrum of PT dinucleotides in the mouse gut microbiome

**Fig. 2:**
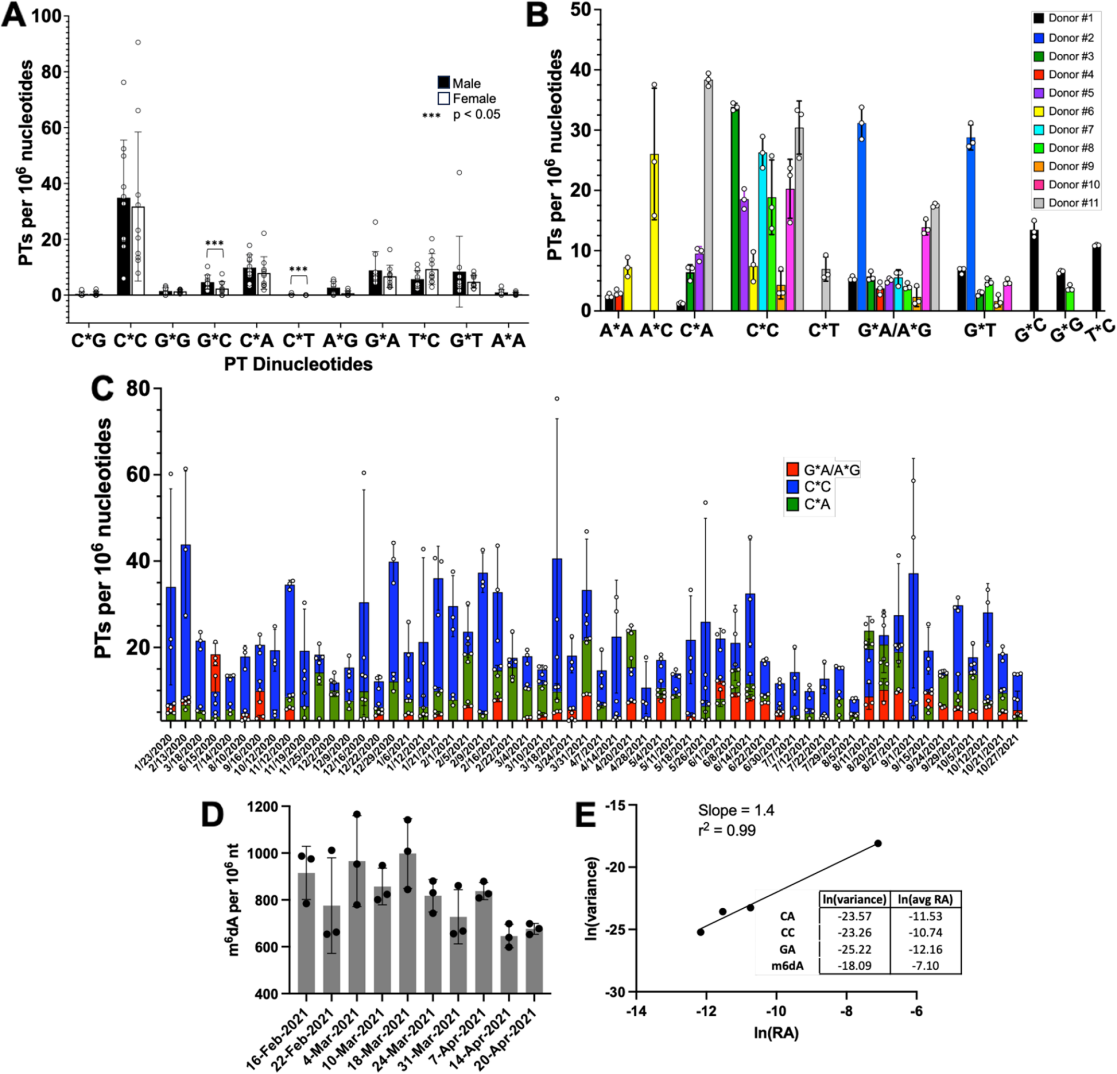
Triple quadrupole LC–MS analysis of mouse and human fecal DNA for PT dinucleotides reveals the presence of PT-containing microbes. **A** Fecal DNA from C57BL/6 mice reveals a uniform spectrum of PT dinucleotides with mostly insignificant differences between males and females. Data represent mean ± SD for 20 mice. Asterisks denote significant differences by the Mann–Whitney test, *p* < 0.05. **B** Fecal PT dinucleotide spectra differ among 11 human donors. Data represent mean ± SD for *N* = 3. **C** Analysis of PT dinucleotides in fecal DNA from donor #5 over 21 months reveals the temporal dynamics of both total PT levels and individual G*A, C*C, and C*A dinucleotides. Data represent mean ± SD for 3 separate DNA isolations from a single fecal sample. The bars representing PT levels for each of the 3 detected PT dinucleotides are superimposed, not stacked. **D** m^6^dA levels in fecal DNA from Donor #5 over 2 months. Data represent mean ± SD for 3 separate DNA isolations from a single fecal sample. **E** Taylor’s Power Law (*V* = am^b^) analysis of temporal dynamics of PT dinucleotides and m^6^dA in Donor #5, where the mean abundance of a species (*m*) in a mixed population fluctuates over time with variance (V) linearly related to m to the power of b. Here a plot of ln(*V*) = ln(*a*) + b*(ln(*m*)) yields *b* = 1.4

As a foundation for human studies, we first assessed the PT landscape in the mouse gut microbiome. With DNA extracted from fecal pellets obtained from individual mice, we quantified PT modifications as PT-bridged dinucleotides by exploiting the resistance of the backbone modification to nuclease P1 [12, 30], which results in a digest containing any of 16 possible PT dinucleotide combinations, all of which have the *Rp* stereochemical configuration of the phosphate [12]. The digestion mixture was analyzed by LC–MS with PT dinucleotides identified relative to chemical standards [12, 30]. The results of such an analysis are shown in Fig. 2A (Supplementary Table S1). It is immediately apparent that the mice all shared a common spectrum of 11 of 16 possible dinucleotides, which might be expected for cage mates of a coprophagic mammal. The only significant difference (*p* < 0.05) between male and female mice involved dG_PT_dC and dC_PT_dT (abbreviated G*C and C*T, respectively). Also telling was the quantity of each PT dinucleotide, which ranged from < 1 to > 90 per 10^6^ total nucleotides. This relatively low abundance compares to PT frequencies of ~ 1 per 10^4^ in individual microbial genomes [30] and is most likely due to the dilution of PT-containing bacteria by the other non-PT species present in the gut microbiome [3, 4]. However, this level of PTs suggests that an average of ~ 5–10% of gut microbes possess PTs, which is consistent with the frequency of genes encoding PT synthesis proteins in > 13,000 individual human microbiome isolates [17].

### Diverse PT dinucleotide spectra in the human gut microbiome

We next compared the mouse data to humans. Analysis of fecal DNA from 11 healthy male and female donors revealed 10 of 16 possible PT dinucleotides (Fig. 2B; Supplementary Fig. S2; Supplementary Table S2A). Here we could not consistently chromatographically resolve A*G and G*A, so we combined the signals for both (denoted G*A/A*G). The results show significant diversity among the donors, with 2 to 7 different PT dinucleotides in each donor (Fig. 2B) and three PT contexts (G*A/A*G, G*T, and especially C*C) broadly shared among the donors. Among the dinucleotides we observed, the PT contexts G*A and G*T can derive from a known double-stranded sequence motif G*AAC/G*TTC modified by the *dnd* gene cluster in microbes such as *E. coli* B7A and *Salmonella enterica* serovar Cerro 8 [12, 30], while C*C was observed in the C*CA motif associated with the *ssp* gene cluster in *Vibrio cyclitrophicus* FF75 [21]. The 10 PT dinucleotide motifs detected here stand in contrast with the preliminary studies of Sun et al. [27] in which only 7 quantifiable PT dinucleotides were detected among 14 individuals; our analysis did not detect T*A but found 4 additional PTs (A*A, C*A, C*T, and G*C). These differences attest to the variation of microbiome PT epigenetics among humans. It is important to point out that the mass spectrometer signals for several PT dinucleotides were confounded by closely-eluting non-PT species in two donors, which warranted confirmation of the LC–MS behavior of all PT contexts for all 11 donors with exact mass analysis using a high-resolution mass spectrometer, as shown in Supplementary Fig. S2. All PT dinucleotide quantification data are based on high-confidence PT dinucleotide signals. This eliminated any uncertainty related to lower-resolution triple-quadrupole analysis, chromatographic peak shifts, or low signal-to-noise ratios.

### Temporal dynamics of PT dinucleotides in the gut microbiome

Four donors contributed fecal samples regularly over periods of weeks to months to allow analysis of the temporal variation in levels of the PT dinucleotides in the gut microbiome. As shown in Fig. 2C and Supplementary Figs. S4–S6 (Supplementary Tables S2B–S2F), while the specific dinucleotide spectra remain roughly constant over time, the total level of all PT dinucleotides in each donor varies significantly on a weekly basis. For example, in donor #5, the presence of the C*A, C*C, G*A/A*G dinucleotides remains constant over the 21-month collection period while the level of total PTs varied by as much as fourfold (Fig. 2C).

To correlate the PT fluctuations with other microbiome epigenetic marks, we measured levels of N^6^-methyl-2’-deoxyadenosine (m^6^dA), a widespread and abundant prokaryotic DNA modification [35, 36], in a subset of samples (10 weeks) from donor #5 and normalized the signals to levels of the canonical nucleosides, as with the PT dinucleotides (Fig. 2D; Supplementary Table S3). The presence of m^6^dA has been confirmed in lower eukaryotes [37], but its presence in mammalian cells is controversial [38] and, if real, would be orders-of-magnitude lower than the levels of 6–10 per 10^4^ nt observed here in fecal DNA (Fig. 2D) [38].

The magnitude of the temporal variation in PT levels in the four donors and the m^6^dA levels in Donor #5 raised the question of parallel behavior in the levels of PT-containing bacteria. The variation cannot be explained by time-dependent changes in PT levels in individual microbes. PT levels do not change significantly as a function of growth conditions [26, 39] likely due to their role in restriction-modification, with reduced PT levels in the face of unchanged restriction activity leading to high levels of DNA strand breaks and reduced fitness [40]. Here we tested the idea that fluctuations in the levels of microbiome DNA modifications followed Taylor’s power law, in which the mean abundance of a species in a mixed population (*m*) will fluctuate over time such that the variance (*V*) follows the equation *V* = am^b^. In human microbiomes, b is consistently > 1, ranging roughly between 1.5 and 2 [41]. For the PT dinucleotides and m^6^dA levels in Donor #5, a plot of ln(*V*) = ln(*a*) + b_*_(ln(*m*)) yields *b* = 1.4 (Fig. 2E; Supplementary Tables S2B, S3, S4), which is consistent with Ma’s definition of a Type III power law extension for mixed-species population spatial aggregation [41]. Further insights into possible shared niches of the various PT-containing microbes can be seen in the time course plots of individual PT dinucleotides for three donors in Supplementary Figs. S4–S6, in which changes in the levels of G*A/A*G and C*C are generally coordinated while those for G*T and G*G are not coordinated with each other or with G*A/A*G and C*C. The latter may thus be in similar growth environments or have similar dependencies. Similarly, the fluctuations in m^6^dA are apparently independent of the PTs. These behaviors beg the question of the identities of PT-containing bacteria in the gut microbiome.

### Metagenomic analysis identifies PT-containing microbes and consensus sequences

To determine the identity of the microbes bearing PTs, we optimized our previously published methods for mapping PT sites using iodine to site-specifically cleave PTs and exploiting the strand breaks to locate PTs in genomes [21, 32]. While the methods proved useful for mapping PTs in pure populations of a single organism, such as the *Escherichia coli* B7A genome with its G*AAC/G*TTC consensus motif [21, 32], they were either too insensitive to analyze mixtures of hundreds of genomes as in fecal DNA or limited to bistranded PTs such as the G*AAC/G*TTC motif, thus ignoring single-strand PTs such as C*CA in *Vibrio cyclitrophicus* FF75 [21, 32]. Here we used an optimized PT mapping method that exploits the T-tailing approach [29, 32] to label iodine-nicked PT sites for subsequent extension, PCR amplification, and NGS sequencing, with attention to maximizing specificity and sensitivity for the complex mixture of genomes in the gut microbiome. With details provided in a separate publication [17], the general concept of “PT-seq” is outlined in Fig. 1C and exploits iodine cleavage of PTs followed by 3′-end poly-(dT) tailing, ddUMP-biotin capping, and subsequent biotin-capture to enrich target DNA, and NGS sequencing library preparation by reverse transcriptase template switching. As a reference gut microbiome genome dataset for metagenomics analyses, we used both sequenced microbe isolates from the human gut in the Broad Institute-OpenBiome Microbiome Library [42, 43] and Metagenome-Assembled Genomes (MAGs) [44] to build a comprehensive custom collection of human gut microbiome (HGM) genomes (13,663 total) that reflected a global, healthy human gut microbiome population (see Methods). The data processing workflow for PT-seq and for parallel shotgun metagenomics of the gut microbiome is shown in Supplementary Fig. S7A.

**Fig. 3:**
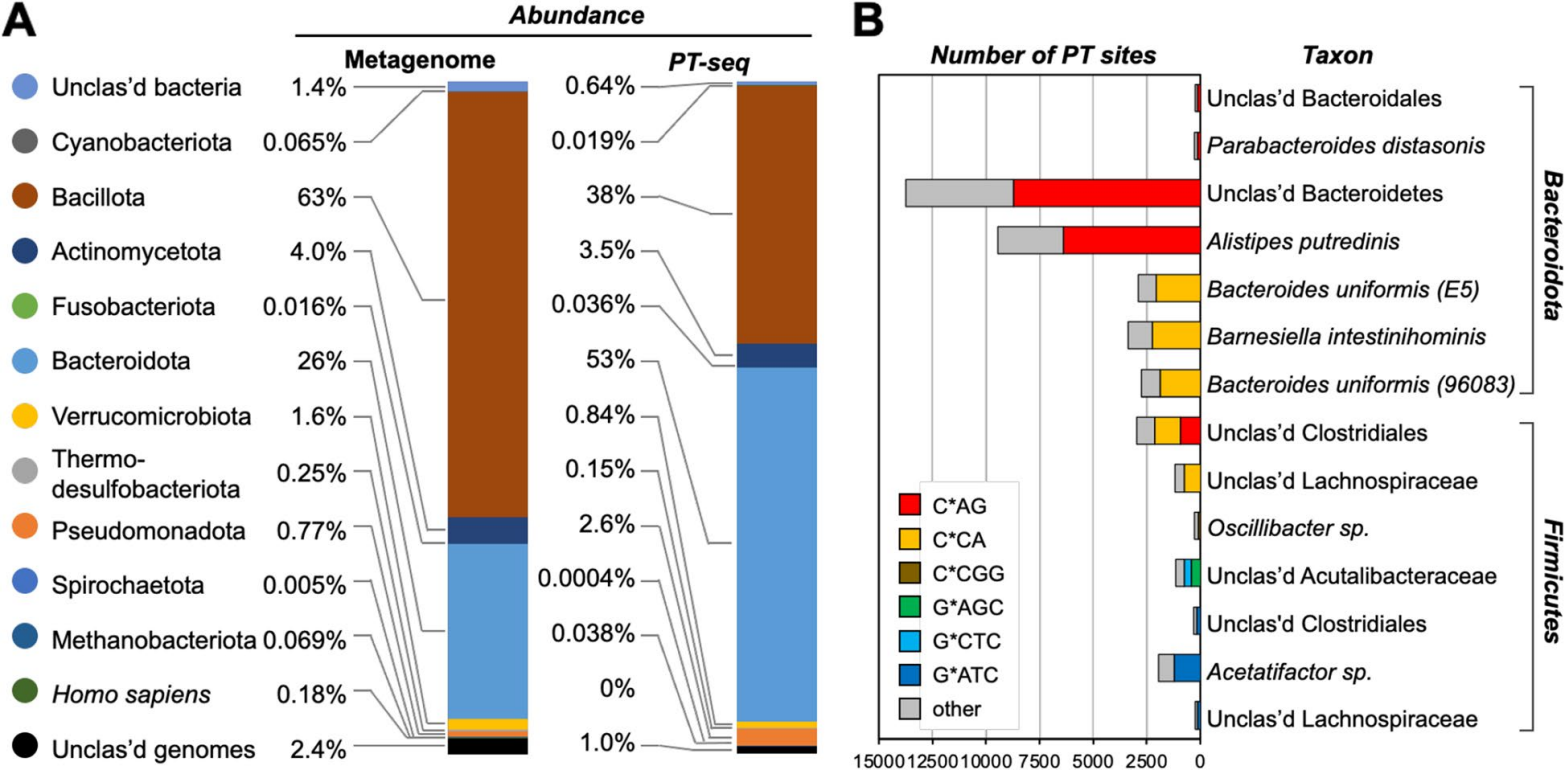
The taxonomic composition of the microbiome and quantification of PT sites. **A** The abundance of the microbiome was estimated by metagenomic sequencing using Kraken2 and Bracken. The phylogenetic composition of microbiome taxa collapsed at the phyla level in the fecal sample (Donor #5). **B** The number of different PT modification motif sites identified by PT-seq in genomes of human gut microbiome. A total of 26,817 sites were detected, with PTs denoted by “*”. Two *B. uniformis strains* (GMbC 3401SE_0218_027_E5 and UHGG 96083) are indicated

This approach was now applied to define the landscape of PT-containing microbes in fecal DNA from Donor #5. We first prepared a reference gut microbiome metagenome for this donor (Supplementary Fig. S7B) from an aliquot of isolated fecal DNA processed without 3′-end terminal blocking or iodine treatment. Taxonomical classification of these shotgun sequencing reads against the custom HGM collection, as well as additional reference databases (see “Methods” section) to identify microorganisms not normally associated with the human gut microbiome (e.g., food), resulted in the classification of 93% of the reads (Supplementary Fig. S7B, Supplementary Table S5A), leaving 7% of the reads as unclassified. Consistent with previously observed human gut microbiome compositions [5, 6], we found that Bacillota was the most abundant phylum, followed by Bacteroidota, Actinomycetota, unclassified genomes, unclassified bacteria, Verrucomicrobiota, and Pseudomonadota (Fig. 3A, “Metagenome”). The number of reads assigned to each genome was used to evaluate the relative abundance of the genome.

We then applied PT-seq to another portion of isolated fecal DNA from Donor #5 and sequenced the library on an Illumina platform. The resulting reads (“PT-seq” in Fig. 3A and Supplementary Fig. S7B) were similarly cross-referenced against the custom HGM collection, resulting in the assignment of 76% of PT-seq reads (Supplementary Fig. S7B, Supplementary Table S5B). We do not know the basis for the 17% difference in assigned reads for the metagenomic reference and PT-seq datasets, but it could be due to a greater proportion of small, unassignable DNA fragments resulting from the additional processing steps of PT-seq or greater resolution of contaminants due to biotin-capture enrichment. Bacteroidota emerged as the dominant phylum of PT-containing bacteria followed by Bacillota, Actinomycetota, and Pseudomonadota (Fig. 3A). These results indicated that the abundance of PT-containing bacteria in the gut microbiome did not simply mirror the taxonomic spectrum of gut bacteria in Donor #5.

### Correlating PT dinucleotides with metagenomic PT consensus sequences

To assign the PT-dinucleotides detected by LC–MS to specific gut microbes, we took advantage of the fact that PT-seq strand breaks occurred site-specifically at PTs and analyzed the sequences at the 5′-ends of the PT-seq reads to identify the consensus sequences of the PT-modifying enzymes in the PT-containing gut bacteria. Here we mapped PT reads to the top 100 genomes found in the HGM custom reference database using Mapper aligner software. The mapping results were further subjected to the Gaussian Mixture Models (GMM) to separate genomes by coverage, median sequencing depth, and dispersion of depth, which resulted in the resolution of the 100 genomes into 3 clusters (Supplementary Fig. S7C). The first cluster contained 28 genomes with coverage > 15% and with a larger median of sequencing depth, so they were likely to represent actual PT capture. Importantly, not all PT-seq sequences were necessarily relevant, since some alignments at this stage likely originated from contaminating DNA fragments from microorganisms that lack PT. In this regard, genomes in the other two clusters with < 15% coverage, a smaller median of sequencing depth, and a larger dispersion were considered less likely to contain PT, since these could also arise from alignments among identical regions shared across many microorganisms..

Using the first cluster of 28 genomes, the consensus motif for PTs in each genome was determined by defining a span of 13-nt centered at each PT site in each read and subjecting these to MEME analysis [45] (Supplementary Table S5C). MEME detected five conserved motifs in 14 PT-containing genomes, including C*AG, C*CA, C*CGG, G*ATC, G*AGC/G*CTC, (Fig. 3B; Supplementary Table S5D). Among them, C*AG and C*CGG have not been reported in an organism previously. We recently confirmed the G*AGC/G*CTC motif in a human gut microbiome isolate *Lachnospiraceae sp.* (GMbC ID 2807EA_1118_063_H5) using PT-seq [17].

To validate these PT-seq-identified PT consensus sequences, we quantified read pileups along each of the 28 genomes with > 15% coverage. Here we first established a minimal read depth for calling a read pileup as a PT site by adjusting the read depth from 1 to 25 and quantifying the total number of read pileups in the genome (Supplementary Fig. S8). As shown in Supplementary Fig. S8, as the read pileup depth increased, the specificity for calling CA, CC, or GA sites, which were noted as sites of PT modification by LC–MS (Fig. 2C), among total pileup sites increased. Reaching a cutoff of 15 converged on 26,507 C*AG, C*CA, C*CGG, G*ATC and G*AGC sites that correlated with the three PT dinucleotides (C*A, C*C, and G*A) (Fig. 2C). This is roughly equivalent to 51 PTs per 10^6^ nucleotides, which is close to our LC–MS average of 43 ± 4 PTs per 10^6^ nt for the same fecal DNA sample analyzed by PT-seq (Fig. 2C, Supplementary data S2B). Therefore, we chose 15 as the optimal read pileup depth that balances sensitivity and specificity for calling sites of PT modification. Using this cutoff, we observed significant read pileups at 70% of putative PT modification sites in 2 out of 14 PT-containing genomes (Supplementary Table S5D). For example, the percentage of pileups at CAG sites was as high as 72.5% in an unclassified Clostridiales genome (GMbC ID 4452YQ_0918_057_F2), while the percentage of CCGG sites was as low as 38% in *Oscillibacter sp.* (genome ID GUT_GENOME141047) (Supplementary Fig. S8; Supplementary Table S5D), with these differences consistent with the frequencies of 3- and 4-nucleotide sequences in a genome, respectively. The observation of less than 100% calling of PTs at a putative modification sequence is consistent with the fact that PT modifications occur at only ~ 10–15% of all possible consensus sequences in a bacterial genome [21]. Interestingly, despite not detecting G*C dinucleotides in our LC–MS analysis of Donor #5 fecal DNA, metagenomic analysis showed that GC sometimes increased in parallel with GA, especially in unclassified Acutalibacteraceae (genome ID GUT_GENOME238522), which would be consistent with a double-stranded G*AGC/G*CTC modification motif as observed in our recent publication [17]. In the genome of unclassified Clostridiales (GMbC ID 4452YQ_0918_057_F2), the specificity of CA and CC sites, which cannot be part of a short palindrome, increased simultaneously (Supplementary Fig. S8; Fig. 3), suggesting two distinct PT modification motifs in the same or similar microorganism(s). We observed 16,317 C*AG, 8,113 C*CA, 107 C*CGG, 1,543 G*ATC, 427 G*AGC, and 310 G*CTC sites (Fig. 3; Supplementary Table S5E), which is equivalent to the following relative abundance: 33.5 C*A, 11.2 C*C, 6.8 G*A and 0.8 G*C. The lack of LC–MS detection of G*C is likely due to the five-fold lower sensitivity for detecting it compared to G*A (Supplementary Fig. S3) and the relatively low level of G*A (Fig. 2C). This discrepancy points to the utility of metagenomics for discovering epigenetic modification sites and the complementarity of metagenomic and mass spectrometric analyses.

## Discussion

The gut microbiome provides an opportunity to characterize polymicrobial interactions in a community of hundreds of prokaryotic species and perhaps thousands of different bacteriophages [2–10]. While the importance of prokaryotic epigenetics is clear in microbial communities, for both defense and regulating gene expression, little is known about the role of microbial epigenetics in the dynamics of the human gut microbiome. Here we focused on DNA phosphorothioate (PT) modifications as widespread prokaryotic and archaebacterial epigenetic marks [12, 21, 26, 30]. The reactivity of PTs with chemical mediators of inflammation, such as neutrophil-derived HOCl, to form lethal DNA strand breaks [26] raises the possibility of major changes in the proportions of PT-containing gut microbiomes in inflammatory bowel disease and other conditions of gut inflammation. As a foundation for studying the potential pathology of PTs, we explored the PT landscape in the healthy gut microbiome using PT-specific metagenomics and mass spectrometry.

Preliminary studies reported the presence of PTs in human fecal DNA but lacked the technology to rigorously quantify the full spectrum of PTs and to identify PT-containing microbes, relying on searches of bacterial genome sequence databases to identify *dnd* gene-containing microbes rather than performing fecal DNA metagenomic analyses [27]. We thus set out to optimize, validate, and apply three technologies for defining the landscape of PT-containing gut bacteria and the behavior of microbiome PT epigenetics (Fig. 1): fecal DNA extraction, LC–MS analysis of PT dinucleotides, and genome-wide site-specific mapping of PTs. For fecal DNA extraction, the tenfold increase in DNA yield that we achieved not only accommodates the larger DNA needs of mass spectrometric analyses—an order of magnitude higher than NGS for metagenomics—but more importantly reduces biases introduced by highly diverse microbial cell wall mechanical properties and complex fecal matrices. Fecal DNA purified with this new method should more faithfully reflect the gut microbiome in health and disease. This increased fecal DNA fidelity combined with the optimized LC–MS method for PT dinucleotide quantification, which now minimizes matrix interferences, further increases the accurate and sensitive of detection of low-abundance PTs. There is still room for improvement in the chromatographic resolution and sensitivity for detecting the 16 PT dinucleotides, with nanoflow chromatography and ever-evolving mass spectrometry technology, such as ion mobility spectrometry [46]. The need for increased LC–MS sensitivity is illustrated by our observation of a PT-seq detection of a G*C-containing sequence in unclassified Clostridiales (Fig. 3) in Donor #5 despite the lack of LC–MS detection of G*C in the same fecal DNA (Fig. 2). As described in a companion publication [17], we revised and optimized previously published NGS PT mapping methods [21, 32] to develop PT-seq (Fig. 1), which allowed sensitive metagenomic analysis of PT-containing microbes and identification of PT consensus sequences.

Using these technologies, we found 10 PT dinucleotides shared by mice and humans (Fig. 2), while C*G was detected only in mice and A*C only in humans, thus accounting for 12 of 16 possible PT dinucleotides. The inability to detect C*G and A*C in their respective hosts or A*T, T*A, T*T, and T*G in either host may simply be due to the low abundance of microbes bearing consensus sequences having these dinucleotide motifs. Indeed, if the detection of T*A by Sun et al. [27] is accurate, then only A*T, T*T, and T*G remain to be identified. The quantitative consistency of the PT dinucleotide spectra among individual male and female mice in our study was not unexpected given observations of highly consistent gut microbe populations in inbred mice due to genetics, shared environments, and coprophagy [47]. However, comparisons among different mouse cohorts in the MIT facility and between mice housed at other institutions will almost certainly reveal differences in the PT spectra in gut microbes given well-established heterogeneity [47].

In the case of humans, the PT dinucleotide spectra were entirely unique for each of the 11 individual donors, with 2 to 7 of 10 different PT dinucleotides detected (Fig. 2). As PT-seq revealed, these dinucleotides are simply fragments of the 3–4 (or longer) consensus sequences for the Dnd, Ssp, and Brex epigenetic families, showing enrichment in Bacteroidota, Bacillota, Actinomycetota, and Pseudomonadota (Fig. 3). These are also the major families of bacteria in the human gut microbiome, with our results shifting the Bacillota/Bacteroidota ratio from 2.42 in all gastro-intestinal microbes to 0.72 in the three seemingly independent groups of PT-containing microbes with C*C, C*A, and G*A/A*G in Donor #5 (Fig. 3). Clearly, we are analyzing the stool microbiome, which most resembles the luminal microbiome and likely does not sample the more controversial mucosal microbiome, especially the crypts [48]. Pseudomonadota genuses Acinetobacter, Delftia, and Stenotrophomonas have been termed the “crypt-specific core microbiome” [49], but Bacteroidota, Bacillota, and nonfermentive Proteobacteria are also found in human colonic crypts [50]. It will be interesting to probe other gut microbiome compartments to assess the populations of PT-containing microbes.

We were initially perplexed by the temporal dynamics of PT dinucleotides and m^6^dA. Visual examination of the PT time courses suggested an almost periodic behavior in PT levels (Supplemental Figs. S4–S6). However, auto-correlation (*r*) analysis of the temporal behavior of the individual PT dinucleotides showed relatively independent changes with no apparent correlation (Supplementary Fig. S6E–L). One possible explanation for the behavior is that PT levels vary significantly within individual microbes due to nutrient (e.g., sulfur) availability or stress. This possibility is ruled out, though, by the fact that bacteria maintain constant levels of PT throughout their genomes under a variety of growth conditions [26, 39] and the fact that reduced PT levels caused by deletion of synthesis genes leads to lethal DNA strand breaks by the remaining restriction system [40]. However, a quantitative test of Taylor’s Power Law revealed that the fluctuations simply reflected the normal dynamics of gut microbes, with less abundant microbes showing the smallest variance (Fig. 2E) [41]. The addition of the much more abundant m^6^dA increased the power of this analysis. Taylor’s constant *b* = 1.4, which falls near the generic expectation of between 1.5 and 2, is entirely consistent with Ma’s definition of a Type III power law extension for temporal sampling of the microbiome [41]. It should be noted that none of the donors adhered to a special diet or strict lifestyle, suffered an illness, or used any antibiotics during the fecal collection period, so the PT compositions and time-dependent behaviors are likely due to the expected vague contributions of genetics [7] and environmental factors [8–10]. That the fluctuations in the PT levels obey expectations for gut microbes is consistent, once again, with the fact that the PT dinucleotides merely represent subsets of the microbiome population.

How do the levels of PT dinucleotides detected here compare to expectations based on the distribution of PT synthesis and restriction genes? The PT-seq results confirm subsets of the gut microbiome possess PT consensus sequences but do not inform about the frequency of those microbes. Jian et al. quantified PT-synthesis *dndA-E* genes in bacteria and archaea in the NCBI genome database and found *dnd* genes in 1.8% of bacteria and 0.7% of archaea, which is close to the 2.7% occurrence of the essential core set of *dndCD* in bacteria in our analysis of 13,000 gut microbiome isolates [17]. However, the occurrence level increases when other families of PT-synthesizing genes are considered, such as *ssp* and BREX, which accounted for an additional 5% of the gut microbe isolates [17]. This total of 7.7% of 13,000 gut microbiome isolates possessing PT-synthesis genes is consistent with the estimate of 5–10% of stool microbes possessing PTs based on (1) the quantity of PT dinucleotides at 1–90 per 10^6^ total nucleotides, (2) an average of ~ 1 PT per 10^4^ nucleotides in individual microbial genomes [30], and (3) dilution of PT-containing bacteria by the other non-PT species present in the gut microbiome [3, 4].

## Conclusions

Here we optimized and applied fecal DNA extraction, LC–MS, and NGS PT-seq technologies to reveal a diverse landscape of PT epigenetics in 5–10% of human gut microbes. Within the context of 11 healthy human fecal donors, LC–MS analysis of limit digests of fecal DNA revealed signature combinations and proportions of the 16 possible PT dinucleotides that reflect bacterial restriction-modification consensus sequences. The PT dinucleotides displayed temporal dynamics consistent with Taylor’s Power Law and the contribution of their host bacteria to the gut population. Application of PT-seq for site-specific metagenomic analysis of PT-containing bacteria in one fecal donor revealed the larger consensus sequences for the PT dinucleotides in Bacteroidota, Bacillota, Actinomycetota, and Pseudomonadota, and further revealed an additional PT consensus sequence not detected by LC–MS. Our results provide a benchmark for understanding the behavior of an abundant and chemically reactive epigenetic mark in the human gut microbiome, with implications for inflammatory conditions of the gut.

## Methods

### Materials

Nuclease P1 (N7000) was purchased from US Biological, Inc and reconstituted according to the manufacturer’s protocol. Calf intestinal alkaline phosphatase (P5521) was from Sigma and reconstituted according to the manufacturer’s protocol. Spin filters (10 kDa) (82031–350) were from VWR. The QIAmp Fast DNA Stool Mini Kit (51604) was purchased from Qiagen. The PowerLyzer PowerSoil (12855) kit was purchased from MO BIO Laboratories, Inc., now part of Qiagen. RNase A (12091021) was purchased from Invitrogen. Ammonium acetate (7.5 M) (A2706) was purchased from Sigma.

### Mouse fecal collection, DNA isolation, and LC–MS analysis of PT dinucleotides

Healthy C57BL/6 mice from The Jackson Laboratory in Bar Harbor, ME were 31–32 days old at the time of collection and weighed from 16 to 23 g. Fecal pellets (3–4) were collected from individual male (10) and female (10) mice under an approved protocol (MIT Committee on Animal Care Protocol 0912–093-15) by removing mice from cages, placing them on a clean bench surface, and collecting freshly excreted fecal pellets. Fresh pellets were placed in a 1.5-mL Eppendorf tube for each mouse and immediately frozen on dry ice, followed by long-term storage at – 125 °C. DNA was isolated from the pellets using the PowerLyzer PowerSoil kit according to manufacturer instructions and all isolations were performed on the same day to reduce experimental bias. Isolated DNA was concentrated under vacuum (SpeedVac, Savant, ThermoFisher), its concentration determined by Nanodrop (ThermoFisher), and the concentration adjusted to 50 ng/μL. For LC–MS/MS analysis, 50 μL (~ 2.5 μg) of DNA were digested with 0.5 U Nuclease P1 (50 °C, 60 min.), followed by 1 U calf intestine phosphatase for 1 h at 37 °C. Samples were filtered with a VWR 10kD MWCO filter to remove proteins and concentrated under vacuum to ~ 30 μL.

PT-linked dinucleotides in the DNA digest (~ 1 µg) were analyzed by LC–MS/MS on a system consisting of an Agilent 6430 ESI-QQQ coupled with an Agilent 1290 HPLC equipped with a diode-array detector (DAD) set to 260 nm absorbance. Chromatographic separation of PT dinucleotides was achieved using a Phenomenex Fusion-Rp column (2.5 µm particle size, 100 Å pore size, 100 mm length, 2 mm i.d.) eluted with a gradient running from 97% buffer A (5 mM NH_4_OAc, pH 5.3) to 9% buffer B (acetonitrile) over 13 min followed by a 1-min column wash with 95% B and re-equilibration with 97% buffer A for 3 min. The QQQ was operated with dynamic multiple reaction monitoring (DMRM) in positive ion mode with the following source parameters: N_2_ gas temperature 350 °C and 10 L/min flow rate, nebulizer pressure 40 psi and capillary voltage 3500 V. HPLC retention times and mass transitions for each of the 16 PT-linked dinucleotides were as detailed in previous publications [12, 26, 30], similar to Table 1 below. Response factors for absolute PT quantification were calculated using external calibration standard curves based on synthetic dinucleotide standards prepared as described previously [12, 26, 30]. Standards were also run in parallel with samples to confirm retention times.

**Table 1:** Mass spectrometry parameters for 16 PT dinucleotides and canonical 2’-deoxyribonucleotides

Compound	Exact mass	Precursor ion	Product ion	Retention time (min)	Collision energy	Cell accelerator voltage
d(A*A)	581.14388	581.1	136.1	12.4–12.5	40	3
d(A*C)	557.13264	557.1	136.1	10.9–11.0	32	3
		557.1	112.1	10.9–11.0	24	3
d(A*G)	597.13879	597.1	152.1	9.61–9.70	48	5
		597.1	136.1	9.70	36	1
d(A*T)	573.13231	572.1	136.1	14.8	32	1
		572.1	127.1	14.8	48	5
d(C*A)	557.13264	557.1	136.1	8.27–8.29	32	1
		557.1	112.1	8.27–8.29	28	3
d(C*C)	533.12141	533.1	112.1	5.31–5.47	16	5
d(C*G)	573.12756	573.1	152.1	5.11–5.31	32	1
		573.1	112.1	5.11–5.31	32	5
d(C*T)	548.12108	548.1	127.1	8.79–8.81	36	5
		548.1	112.1	8.79–8.81	28	5
d(G*A)	597.13879	597.1	152.1	9.61–9.70	28	3
		597.1	136.1	9.61–9.70	32	1
d(G*C)	573.12756	573.1	152.1	7.64–7.65	32	5
		573.1	112.1	7.64–7.65	40	3
d(G*G)	613.13371	613.1	152.1	7.14–7.20	36	3
d(G*T)	588.12722	588.1	152.1	11.5–11.6	32	5
		588.1	127.1	11.5–11.6	40	3
d(T*A)	573.13231	572.1	136.1	12.92	28	3
		572.1	127.1	12.9	44	3
d(T*C)	548.12108	548.1	127.1	10.1–10.2	44	1
		548.1	112.1	10.1–10.2	24	5
d(T*G)	588.12722	588.1	152.1	9.18–9.20	32	5
		588.1	127.1	9.18–9.20	48	1
d(T*T)	563.12074	563.1	127.1	14.4	40	5
dA	252.10912	252.1	136.1	5.23	12	1
dC	228.09788	228.1	112.1	1.45	16	1
dG	268.10403	268.1	152.1	2.71	4	3
dT	243.09755	243.1	127.1	3.22	12	1

### Collection of human feces

The collection of human fecal matter from healthy adult volunteers was performed under a protocol approved by the MIT Committee on Use of Human Subjects (Protocol 2,306,001,007). Donors were provided with a kit consisting of a large weigh boat to assist collection, a plastic micro spatula for manipulation, a 50-ml centrifuge tube for storage, a pair of large nitrile gloves, a re-sealable zipper plastic bag for organization and temporary ice storage, and a small paper bag for concealment. Donors were instructed to drop off fecal samples for processing immediately, place the sample at 4 °C for up to 3 h prior to delivery, or store the samples at − 20 °C. All samples were processed within 24 h of collection in any event.

### Optimization of protocol Q for DNA isolation from feces

To enhance the yield of DNA from fecal extraction and ensure an unbiased population of microbial genomes, we optimized each step of the International Human Microbiome Standards (IHMS) IHMS_SOP 06 V3: Standard operating procedure for fecal samples DNA extraction, Protocol Q [31].

#### Amount of human fecal material

Varying amounts of fecal material were weighed into a 15-mL tube (200, 400, or 800 mg). To the tube was added 4 mL of sterile PBS (10 mM, pH 7.4). The mixture was homogenized using a Qiagen tissue homogenizer at full speed for 1 min. Two hundred microliters of this mixture was used for the DNA isolation and the isolation procedure proceeded as published in IHMS Protocol Q (IHMS_SOP 06_v2 from 4/12/2015).

#### Bead beating step

Varying amounts of fecal material were weighed into a 2-mL tube (100, 150, or 200 mg). Samples were prepared in duplicate, one set to be processed with beads and one set to be processed without beads. The DNA isolation followed IHMS Protocol Q as published in IHMS_SOP 06_v2 from 4/12/2015 with several modifications: After the addition of 1 mL of InhibitEx buffer to the 2 mL tube containing fecal material, the sample was homogenized on a FastPrep homogenizer for a total of 2 cycles, 45 s per cycle at a speed setting of 6.0.

#### PT dinucleotide stability following timed heating at 95 ˚C

Here we added 20 µg (200 µL) of purified genomic DNA isolated from *E. coli B7A* to a 2 mL tube. Our DNA isolation protocol was carried out exactly as described above but with varying incubation times at 95 °C. Heating was carried out for either 5, 10, 15, or 20 min. After completion of the protocol, the DNA was digested into a mixture of mononucleosides and PT-containing dinucleotides using NP1 and CIAP as described above. PT dinucleotide levels were quantified by LC–MS as described above.

### Extraction of bacterial DNA from human fecal samples using optimized Protocol Q

Fecal material (180–220 mg) was combined with 0.1 mm (0.3 g) and 0.5 mm (0.3 g) Zirconia beads and 1 mL of InhibitEx buffer from the QIAmp Fast DNA Stool Mini Kit in a 2-mL tube. The mixture was processed on a FastPrep Cell Disruptor for 45 s using a speed setting of 6.0 (2 total cycles of bead beating). The mixture was then incubated at 95 °C for 15 min. The mixture was again homogenized on a FastPrep Cell Disruptor for 45 s using a speed setting of 6.0 (2 total cycles of bead beating). Solid material was pelleted via centrifugation at 4 °C for 5 min at 16,100 × *g*. The supernatant was transferred to a new 2-mL tube and kept on ice. InhibitEx buffer (300 µL) was added to the pellet and the mixture was again homogenized on a FastPrep Cell Disruptor for 45 s using a speed setting of 6.0 (2 total cycles of bead beating). Solid material was pelleted via centrifugation at 4 °C for 5 min at 16,100 × *g*. The supernatant was pooled with the previous supernatant. Ammonium acetate (260 µL of 7.5 M) was added to the pooled supernatants, and the solution was vortexed and incubated on ice for 5 min. Precipitate was pelleted by centrifugation at 4 °C for 10 min at 16,100 × *g*. The supernatant was removed and aliquoted into separate 2 mL tubes (650 µL each). One volume (650 µL) of isopropanol was added to each aliquot, and the mixtures were vortexed and incubated on ice for 30 min. DNA was pelleted by centrifugation at 4 °C for 15 min at 16,100 × *g*. The supernatant was discarded and pellets were gently washed with 70% ethanol (500 µL) by inverting 3 times before centrifugation at 16,100 × *g* at 4 °C for 15 min. Excess supernatant was removed by aspiration, pellets were air dried for 10 min, and then solubilized in 100 µL Tris–HCl (10 mM, pH 8.0). The solutions were pooled, combined with RNase A (2 µL of 20 mg/mL), and incubated at 37 °C for 10 min.

Proteinase K (15 μL) was added to a 1.5-mL tube before the addition of the resuspended precipitated DNA (200 μL) and Buffer AL (200 μL) from the Qiagen Fast DNA Stool Kit. The mixture was vortexed (15 s) and incubated at 70 °C for 10 min. Ethanol (200 μL) was added to the mixture and the entire mixture (600 μL) was added to a QIAmp spin column, centrifuged at 16,100 × *g* for 1.5 min, and the flow-through was discarded. The column was then washed with Buffer AW1 (600 μL), centrifuged at 16,100 × *g* for 1.5 min, and the flow-through was discarded. The column was then washed twice with Buffer AW2 (600 μL) as above. The column was centrifuged at 16,100 × *g* for 3 min to remove residual buffer. Pre-heated Tris–HCl (100 μL, 10 mM, pH 8.0) was applied to the column, incubated for 1 min, and centrifuged at 16,100 × *g* for 1.5 min to elute the DNA. The yield and purity of DNA were assessed by Nanodrop. DNA was stored at − 20 °C.

### Digestion of DNA for LC–MS/MS analysis of PT dinucleotides

DNA (20 μg, 78 μL) was incubated with Nuclease P1 (1.5 U, 3 μL) in 30 mM ammonium acetate pH 5.3 and 0.5 mM ZnCl_2_ (90 μL total reaction volume) for 2 h at 55 °C. The reaction mixture was diluted with Tris–HCl (~ 100 mM final concentration, pH 8.0, 9 μL) and incubated with calf intestinal alkaline phosphatase (51 U, 3 μL) for 2 h at 37 °C. Enzymes were removed by passing the mixture through a VWR 10 kDa spin filter with centrifugation at 12,000 × *g* for 12 min. The solution was lyophilized to dry and resuspended in H_2_O (50 μL).

### LC–MS/MS analysis of PT dinucleotides

Synthetic PT DNA dinucleotides or Nuclease P1 hydrolyzed DNA were analyzed by LC–MS/MS on an Agilent 1290 series HPLC system equipped with a Synergi Fusion RP column (2.5 μm particle size, 100 Å pore size, 100 mm length, 2 mm inner diameter) and a DAD. The HPLC was coupled to an Agilent 6490 triple quadrupole mass spectrometer. The column was eluted at 0.35 mL/min at 35 °C with a linear gradient of 3–9% solvent B (acetonitrile) in solvent A (5 mM ammonium acetate pH 5.3) over 15 min. The column was then washed with 95% solvent B for 1 min, and then re-equilibrated with 97% solvent A for 3 min. Canonical deoxyribonucleosides that eluted from the column were quantified by their 260 nm absorbance with the DAD. PT-containing dinucleotides were identified and quantified by tandem quadrupole mass spectrometry with electrospray ionization operated with the following parameters: N_2_ temperature, 200 °C; N_2_ flow rate, 14 L/min; nebulizer pressure, 20 psi; capillary voltage, 1800 V; and a fixed fragmentor voltage, 380 V. For product identification, the mass spectrometer was operated in positive ion polarity using dynamic multiple reaction monitoring (DMRM) modes with the conditions tabulated below.

### High resolution mass spectrometry

Synthetic PT dinucleotides (2 pmol per 10 μL injection) or Nuclease P1 hydrolyzed RNA (4 μg per 10 μL injection) were analyzed on a Dionex Ultimate 3000 UHPLC system equipped with a Synergi Fusion RP column (2.5 μm particle size, 100 Å pore size, 100 mm length, 2 mm inner diameter). The HPLC was coupled to a Thermo Fisher Q Exactive Hybrid Quadrupole-Orbitrap mass spectrometer. The column was eluted at 0.35 mL/min at 35 °C with a linear gradient of 3–9% acetonitrile in 97% solvent A (5 mM ammonium acetate pH 5.3) over 15 min. The column was washed with 95% acetonitrile in solvent A for 1 min, and initial conditions were regenerated by equilibrating the column with 97% solvent A for 3 min. High-resolution mass spectra for the PT-containing dinucleotides were obtained by hybrid quadrupole-Orbitrap mass spectrometry with the following parameters: sheath gas flow rate, 50 L/min; aux gas flow rate, 15 L/min; sweep gas flow rate, 3 L/min; spray voltage, 4.20 kV; and capillary temperature, 275 °C. For product identification, the mass spectrometer was operated in positive ion polarity using targeted single ion monitoring mode with the conditions tabulated in Table 1.

### Raw mass spectrometry data processing and statistical analysis

Raw data were processed using either Agilent MassHunter Qualitative Analysis software for samples analyzed on the Agilent 6490 instrument or Thermo Xcalibur Qual Browser for samples run on the QExactive instrument. Only signals with distinct peak shapes, determined by visual inspection, were considered for data analysis. Each PT dinucleotide signal was correlated to a response factor from runs using calibration standards. Absolute PT levels per 10^6^ nucleotides were calculated based on the estimated amount of injected canonical nucleosides using UV signals at 260 nm in correlation with UV calibration curves.

### Metagenomic sequencing

For shotgun metagenomics, 5 µg DNA was extracted from a Donor #5 fecal sample using optimized protocol Q and submitted to Novogene (UC Davis, CA, USA). After QC, DNA was fragmented and subjected to end repair and phosphorylation. Next, A-tailing and adaptor ligation were performed in a PCR-free method. Finally, 150 bp paired-end sequencing was performed with the Illumina Novaseq 6000.

### Defining reference genomes for metagenomic sequencing

The first step in mapping PT modifications was to define a reference human gut microbiome for comparisons with our human gut microbiome sequences, using both microbe isolates and Metagenome-Assembled Genomes (MAGs). We designed a comprehensive custom collection of human gut microbiome (HGM) genomes that reflect a global, healthy human gut microbiome population. This reference collection contains 3632 genomes of gut bacterial isolates from the Broad Institute-OpenBiome Microbiome Library (BIO-ML) [42], 5387 genomes of gut bacterial isolates from the Global Microbiome Conservancy (GMbC) collection [43], and 4644 genomes of gut microbial isolates and MAGs from the Unified Human Gastrointestinal Genome (UHGG) collection [44]. The 9019 genomes from GMbC and BIO-ML were filtered using drep (v2.5.4) with 99% average nucleotide identity[51]. We taxonomically classified sequencing reads against the custom reference database (50,67 genomes total) using Kraken2 (v2.1.3) [52] and Bracken (v2.8) [53].

### Library preparation for PT-seq

 As detailed elsewhere (DOI: 10.1101/2024.06.03.597111), optimized signals for PT-seq were achieved in part by employing 4 cycles of denaturation, dephosphorylation, followed by dideoxy-NTP end capping to block pre-existing strand-break sites. Each cycle started by denaturing at 94 °C for 2 min and immediately cooling down on ice for 2 min. The initial dephosphorylation reaction was in a mixture (50 μL) containing 5 μL of terminal transferase buffer (NEB Tdt Reaction Buffer, Catalog # M0315S), 1 μL of shrimp alkaline phosphatase (rSAP, NEB Catalog # M0371S), and 10 μg of fragmented DNA, with incubation at 37 °C for 30 min to remove phosphate at 3′ end of the strand-breaks. The phosphatase was then inactivated by heating at 65 °C for 10 min. After cooling, 1 μL of Tdt Reaction Buffer, 6 μL of CoCl_2_ (0.25 mM), 2 μL of ddNTPs (2 mM each, TriLink), and 1 μL of terminal transferase (20 units, NEB Catalog #M0315S) was added to the reaction with incubation at 37 °C for 1 h to block any pre-existing strand-break sites. Blocking cycles were repeated 3 times, in which fresh reagents were added in each cycle (0.25 μL of Tdt Reaction Buffer, 0.25 μL of CoCl_2_, 2 μL of ddNTPs, and 0.5 μL of Tdt). The blocked DNA was purified using a DNA cleanup kit (Zymo Catalog # 11-304C).

For iodine cleavage, 32 μL of the blocked DNA was incubated with 4 μL of 500 mM Tris–HCl pH 10.0 and 4 μL of iodine solution (50 mM, Fluka, Catalog # 318981–100) at room temperature for 5 min. Then, the reaction product was purified using two b columns (QIAGEN Catalog #63206) to remove salts and iodine. The purified DNA was denatured by incubating at 94 °C for 2 min and cooling on ice for 2 min and then incubated with 4 μl of H_2_O, 5 μL of NEB rCutsmart buffer, and 1 μL of rSAP (50 μl reaction) to remove 3′-phosphates arising from iodine cleavage. After incubation at 37 °C for 30 min and 65 °C for another 10 min, the product was incubated with 1 μl of dTTP (1 mM, NEB Catalog # N443S), 1 μL of Tdt buffer, 6 μL of CoCl_2_, 1 μL of Tdt and 1 μL of H_2_O. After incubation at 37 °C for 45 min, the dTTP was removed by DyeEX columns.

DNA (60 μL) was then incubated with 7.8 μL of Tdt buffer, 7.8 uL of CoCl_2_, 1 μL of ddUTP-biotin (1 mM, Jena Bioscience NU-1619-BIOX-S), 1 μL of Tdt and 1 μL of H_2_O at 37 °C for 1 h to terminate the T-tails by ddUTP-biotin. After cleaning with DyeEX columns, the DNA was diluted in 500 μL of H_2_O and fragmented by probe sonication as described elsewhere (DOI: 10.1101/2024.06.03.597111). The DNA fragments were mixed with 10 μL of streptavidin-coated magnetic beads (NEB Catalog # S1421S) and 500 μL of binding buffer (5 mM Tris–HCl, pH 7.5, 1 M NaCl, 0.5 mM EDTA) and incubated on a shaker at ambient temperature for 1 h. The beads were pulled down by a magnetic and washed 3 times with 100 μL of binding buffer. After discarding the supernatant, the beads were resuspended in 20 μL of H_2_O.

The beads were then ready for Illumina library preparation with SMART ChIP-seq kit (Takara, Catalog # 634865) by following the manufacturer’s protocol. The final step of PCR was performed using the Illumina primers provided in the ChIP-seq kit and 12 cycles were used for amplification. The PCR product with a unique sequencing barcode was submitted to an Illumina MiSeq instrument for 150 bp paired-end sequencing.

### Data analysis

Adapters were removed using bbduk from BBtools (v39.01) (sourceforge.net/projects/bbmap/), (with parameters ktrim = r *k* = 18 hdist = 2 hdist2 = 1 rcomp = f mink = 8 qtrim = r trimq = 30 for R1, ktrim = r *k* = 18 mink = 8 hdist = 1 rcomp = f qtrim = r trimq = 30 for R2). T-tails were removed using bbduk with parameters ktrim = r k = 15 hdist = 1 rcomp = f mink = 8 for R1 and ktrim = l *k* = 15 hdist = 1 rcomp = f mink = 8 for R2. PhiX were removed using bbduk with parameters *k* = 31 hdist = 1.

We collected 3632 genome sequences from the BIOML[42] and 5387 genome sequences from GMbC [43]. We filtered genomes at an estimated species level (ANI ≥ 99%) using dRep v2.5.4 [51] with the options: -sa 0.99. We collected 4644 genome sequences from the UHGG collection [44]. For metagenomic sequencing data, the trimmed reads were classified against the custom library built with filtered human gut microbiome sequences from BIOML, GMbC, and UHGG using Kraken2 v2.1.3 [52] and Bracken v2.8 [53]. The relative abundances of genomes were calculated using the number of reads assigned.

For PT-seq, we mapped the resulting reads to 100 genomes with the most reads assigned using Mapper v1.1-beta04 (github.com/mathjeff/Mapper). If a read maps to multiple positions with the optimal alignment quality, we output all alignment results and computed depth to each position as 1/n (*n* = number of optimal aligned positions). To separate genomes that contained PT (Fig. S7C, cluster 1), were missing PT entirely (Fig. S7C, cluster 2), or potentially contained false positive (Fig. S7C, cluster 3), the resulting coverage, depth, and dispersion of depth were subjected to clustering using the scikit-learn Gaussian Mixture Models (GMM) with a custom python script. Twenty-eight genomes in the cluster (Fig. S7C, cluster 1) with more than 15% coverage were then subjected to consensus motif detection. Using custom scripts, the 13-nt sequences centered at 5′-ends of read pileups were retrieved from vcf files generated by Mapper. With an incrementing cutoff of the depth of read pileups from 1 to 25, the number of pileups at each NN nucleotide site was counted using homemade scripts, and the motifs were analyzed using MEME v5.2.0 [45] with parameters: -dna -objfun classic -nmotifs 5 -mod zoops -evt 0.05 -minw 3 -maxw 6 -markov_order 0 -nostatus -oc. The equivalent PTs were calculated by the number of read pileups derived from the genome size and normalized to the relative abundance.

### Statistical analysis

LC–MS data for mouse PT dinucleotides shown in Fig. 2A were determined to fail normality distribution for each group (male and female) using several tests, including D’Agostino and Pearson, Anderson–Darling, Shapiro–Wilk, and Kolmogorov–Smirnov. Therefore, statistical significance was determined using a non-parametric Mann–Whitney test for unpaired groups that do not follow a normal distribution. The results of this analysis are shown in Table 2. A *p*-value threshold of 0.05 was used to determine the statistical significance of the data.

**Table 2:** Mann–Whitney tests for mouse data shown in Fig. 2A

PT dinucleotides	*p-*value	Mean rank of male	Mean rank of female	Mean rank difference	Mann–Whitney *U*	*q*-value
C*G	0.87	10.2	10.8	− 0.6	47	0.97
C*C	0.53	11.4	9.6	1.8	41	0.84
G*G	0.84	10.8	10.2	0.6	47	0.97
G*C	0.04	13.2	7.8	5.4	23	0.23
C*A	0.28	12	9	3	35	0.52
C*T	0.03	13	8	5	25	0.23
A*G	0.10	12.7	8.3	4.4	28	0.23
G*A	0.63	11.2	9.8	1.4	43	0.88
T*C	0.11	8.3	12.7	− 4.4	28	0.23
G*T	0.97	10.4	10.6	− 0.2	49	0.98
A*A	0.09	12.75	8.25	4.5	27.5	0.23

## Supplementary Information

Supplementary Material 1.

Supplementary Material 2.

Supplementary Material 3.

Supplementary Material 4.

Supplementary Material 5.

Supplementary Material 6.

Supplementary Material 7.

## Data Availability

Raw metagenomic sequencing data and raw PT-seq data have been uploaded to the NCBI SRA database with BioSample ID SAMN44015141 and SAMN39420940, respectively, and BioProject ID PRJNA1006039. The custom scripts used in the study is available at https://github.com/dedonlab/published_scripts. Raw LC-MS data have been deposited to the ProteomeXchange Consortium via the PRIDE [54] partner repository with the project accession # PXD051389.
