# Supplementary material for: Temporal dynamics and metagenomics of phosphorothioate epigenomes in the human gut microbiome
Source: Microbiome. 2025 Mar 24;13:81. doi: 10.1186/s40168-025-02071-4 (PMC11931770; doi:10.1186/s40168-025-02071-4)
Supplement: Supplementary file 8 — Supplementary Material 7. [file 40168_2025_2071_MOESM7_ESM.docx]

**Supplementary Information for**

**“Temporal dynamics and metagenomics of phosphorothioate epigenomes in the human gut microbiome”**

Shane R Byrne, Michael S DeMott, Yifeng Yuan, Farzan Ghanegolmohammadi, Stefanie Kaiser, James G. Fox, Eric J. Alm and Peter C. Dedon

**Supplementary Figures:**

**Fig. S1.** Optimization of Protocol Q for fecal DNA extraction.

**Fig. S2.** Exact mass confirmation of PT dinucleotides using Orbitrap for donors #1-#11.

**Fig. S3**. LC-MS calibration curves for PT dinucleotides for donor #5.

**Fig. S4.** PT dinucleotide time course for donor #7 for **(A)** total PTs, **(B)** G*A, and **(C) C*C**.

**Fig. S5.** PT dinucleotide time course for donor #8 for (**A**) total PTs, (**B**) G*T, (**C**) G*G, (**D**) G*A, and (**E**) C*C.

**Fig. S6**. PT dinucleotide time course for donor #9 for (**A**) total PTs, (**B**) G*T, (**C**) G*A, and (**D**) C*C. (**E-L**) Test of periodicity for each PT dinucleotide (C*A, C*C, G*A, and total) from donor #5.

**Fig. S7**. Workflow for processing of metagenomic sequencing and PT-seq data.

**Fig. S8**. The number of read pileups (y-axis) with an increasing depth cutoff (x-axis) in 28 genomes with >15% coverage.

**Fig. S9**. The percentage of read pileups at each of 16 possible dinucleotide sites.

**Supplementary Tables – all as separate Excel spreadsheets**

**Table S1.** PT dinucleotides in fecal DNA from mice. This source data is associated with **Figure 2A.**

**Table S2A**. PT dinucleotides in fecal DNA from 11 human donors. This source data is associated with **Figure 2B.**

**Table S2B**. Time course of PT dinucleotide levels in Donor #5. This source data is associated with **Figure 2C, 2D, 2E**.

**Table S2C**. Time course of PT dinucleotide levels in Donor #7. This source data is associated with **Figure S4**

**Table S2D**. Time course of PT dinucleotide levels in Donor #8. This source data is associated with **Figure S5**.

**Table S2E**. Time course of PT dinucleotide levels in Donor #9. This source data is associated with **Figure S6**.

**Table S2F**. Calibration curves for PT dinucleotides and canonical nucleosides. This source data is associated with **Figure S3**.

**Table S3**. Time course of m^6^dA in Donor #5. This source data is associated with **Figures 2C** and **2D**.

**Table S4.** Taylor’s power law analysis of PT dinucleotide levels in Donor #5. This source data is associated with **Figure 2E**.

**Table S5.** PT consensus sequence metagenomics.

**Fig. S1.** Optimization of Protocol Q for fecal DNA extraction. (**A**) Comparisons of DNA yield and purity for the original, unoptimized Protocol Q (red) and two independent repetitions of the QIAamp Fast DNA Stool Kit (black and blue) with the same human fecal sample. (**B**) DNA yield relative to increasing amounts of fecal material in the same 200 μL volume. The red asterisk denotes the original conditions from Protocol Q, in which only 5% of 20-fold diluted fecal matter was used for DNA purification. The revised method skips the dilution step and uses the entire fecal sample for DNA purification. The yield of DNA from 200 mg of feces thus increased from 2.4 μg to >30 μg. (**C**) DNA yield from human feces when using glass beads (red) or no glass beads (blue) in the revised Protocol Q. (**D, E, F**) Comparison of DNA yield (**D**), A_260_/A_230_ ratio (**E**), and A_260_/A_280_ ratio (**F**) with the QIAamp Fast DNA Stool kit (blue) and the optimized Protocol Q (red). DNA was extracted from human fecal samples from 6 donors using the two different protocols. (**G**) Effect of heat on the stability of phosphorothioates in PT-bearing genomic DNA isolated from *E. coli* B7A and spiked into human feces. PT dinucleotides were analyzed in nuclease-digested fecal DNA analyzed by LC-MS. (**H**) HPLC elution profile (UV detection) of nuclease digests of human fecal DNA isolated using the QIAamp Fast DNA Stool kit (upper) and the optimized Protocol Q (lower).

**Fig. S2.** Exact mass confirmation of PT dinucleotides using Orbitrap for donors #1-#11. For each donor, from the left, the first and second columns show extracted ion chromatograms for the putative PT dinucleotide and the relevant standard, respectively, and the third and fourth columns show the mass spectra of the putative PT dinucleotide and the relevant standard, respectively, within 10 ppm.

**Fig. S2.** Continued.

**Fig. S2.** Continued.

**Fig. S2.** Continued.

**Fig. S2.** Continued.

**Fig. S2.** Continued.

**Fig. S3**. LC-MS calibration curves for PT dinucleotides for donor #5. Calibration curves for the PT dinucleotides C*A **(A)**, C*C **(B)**, G*C **(C)** and G*A **(D)** were generated by plotting the amount of each PT dinucleotide synthetic standard that was injected onto the LC-MS against the measured abundance for the indicated MRM transition by LC-MS. **(E)** Calibration curves for the 4 canonical deoxyribonucleosides were generated by plotting the amount of each deoxyribonucleoside synthetic standard that was injected onto the LC-MS against the measured UV signal at 260 nm using the diode array detector on the LC-MS. A line of best fit was constructed for each calibration curve, along with the equation describing the line of best fit and the coefficient of determination (R^2^).

**Fig. S4.** PT dinucleotide time course for donor #7 for **(A)** total PTs, **(B)** G*A, and **(C) C*C**. Data represent mean ± SD for N=3.

**Fig. S5**. PT dinucleotide time course for donor #8 for (**A**) total PTs, (**B**) G*T, (**C**) G*G, (**D**) G*A, and (**E**) C*C. Data represent mean ± SD for N=3.

**Fig. S6**. PT dinucleotide time course for donor #9 and analysis of periodicity in PT levels. Donor #9 time course for (**A**) total PTs, (**B**) G*T, (**C**) G*A, and (**D**) C*C. Data represent mean ± SD for N=3. (**E-L**) Test of periodicity for each PT dinucleotide (C*A, C*C, G*A, and total) from donor #5. (**E, G, I, K**) Mean values of three replicates for C*A (**E**), G*A (**G**), C*C (**I**), and total PTs (“sum”; **K**) were *log*-transformed. (**F, H, J, L**) The normality of the transformed data for **E**, **G**, **I**, and **K** were checked using the Shapiro-Wilk test (“shapiro.test” function of R “stats” package) at P < 0.05. The “acf” function of R “stats” package was used to compute autocorrelation. Note: a negative correlation means that relative pairs move in opposite directions, while a positive correlation means they move in the same direction.

**Fig. S7.** Processing of metagenomic sequencing and PT-seq data. (A) The scheme of investigation of PT landscape of human gut microbiome. Metagenomic sequencing reads were assigned to HGM genomes. PT-seq reads were mapped to the most abundant 100 genomes using Mapper v1.1-beta04. The genomes with above 15% coverage were subjected to MEME consensus motif detection and pileup site analyses, in which the cutoff of read pileup depth was determined by the quatification of PT dinucleotides using LC-MS. (B) The read classification of metagenomic sequencing (reference) and PTseq of human gut microbiome genomes of donor #5. (C) The clustering of the most abundant 100 genomes for PTseq. PTseq reads were mapped to 100 genomes with the most read assigned by Bracken using Mapper v1.1-beta04 (github.com/mathjeff/Mapper). The coverage, median depth and dispersion of depth were plotted and clustered using GMMs. Genomes in cluster 1 were subjected to pileup site analyses.

**Fig. S8.** The number of read pileups (y-axis) with an increasing depth cutoff (x-axis) in 28 genomes with >15% coverage. Each graph depicts 1 of the 28 genomes. The number of read pileups decreases with increasing depth cutoff in a negative log-like manner. Reaching a cutoff of 15 converged on 26,507 C*AG, C*CA, C*CGG, G*ATC and G*AGC sites that correlated with the LC-MS levels of three PT dinucleotides (C*A, C*C, and G*A).

**Fig. S9.** The percentage of read pileups at each of 16 possible dinucleotide sites. The percentage (y-axis) as a function of read pileup depth cutoff (x-axis) in 28 genomes with >15% coverage. Each graph depicts 1 of the 28 genomes.
